# Pharmacological Evaluation of *Amorphophalli rhizoma* to Inhibit the Progression of Estrogen Receptor^+^ (ER^+^) Breast Cancer by Modulating the PI3K/AKT Cell Signaling Pathway

**DOI:** 10.2174/0115680096360994250106082347

**Published:** 2025-03-05

**Authors:** Hailong Li, Qinghong Yu, Jiaqing Song, Haining Ding, Yian Chen, Ying Jin, Hongting Wu, Liaqat Hussain, Xiufei Gao

**Affiliations:** 1 School of Green Intelligent Pharmaceutical, Zhejiang Guangsha Vocational and Technical University of Construction, Dongyang, Zhejiang, China;; 2 First Clinical Medical College, Zhejiang Chinese Medical University, Hangzhou, Zhejiang, China;; 3 Department of Pharmacology, Faculty of Pharmaceutical Sciences, Government College University, Faisalabad, Pakistan;; 4 Department of Breast Surgery, The First Affiliated Hospital of Zhejiang Chinese Medical University, Zhejiang Provincial Hospital of Chinese Medicine, Hangzhou, Zhejiang, China

**Keywords:** Breast cancer, *Amorphophalli rhizoma*, estrogen receptor-positive, PI3K/AKT pathway

## Abstract

**Introduction:**

Breast Cancer (BC) is one of the most prevalent malignant tumors in women. The incidence of estrogen receptor-positive (ER^+^) breast cancer is as high as 70%, and it is increasing. *Amorphophalli rhizoma* (APR) has the potential to be used in breast cancer.

**Aims:**

The objectives of the present study were to explore the impact of different APR extracts on the proliferation, migration, and invasion of ER^+^BC and to investigate their possible mechanism at the molecular level.

**Methods:**

Various extracts of APR were prepared in different solvents, such as petroleum ether, ethyl acetate, n-butanol, and water. ER^+^ T47D breast cancer cell lines were acquired and utilized to assess the effect of APR extracts on ER^+^ BC. Cell viability was assessed using the cell counting kit8 (CCk8) method, while anti-invasive and migratory effects were examined by transwell and wound healing assay. All the extracts were initially screened, and the ethyl acetate fraction (APR-EA) was found to be the most effective. Ultra High-Performance Liquid Chromatography (UHPLC) of APR-EAE extract revealed the presence of various phytochemicals, such as succinic acid, 2-methoxy resorcinol, penicillic acid, morphine, salicylic acid, α-linolenic acid, and linolenic acid. Flow cytometry, western blot, and immunohistochemistry were used to explore molecular mechanisms.

**Results:**

APR-EA demonstrated anti-proliferative, anti-migratory, and anti-invasive effects on the ER^+^ T47D cell line. Thus, APR-EAE might inhibit the expression of P-PI3K/PI3K and P-Akt/Akt proteins, which subsequently represses the expression of ERα. This inhibition affects the downstream expression of the proteins CDK4 and Bcl-2, which are linked to cell growth and apoptosis.

**Conclusion:**

Additionally, APR-EA might increase the expression of P21 and Bax proteins, which are associated with cell cycle arrest and apoptosis. Overall, these effects contribute to the anti-ER^+^ breast cancer properties of APR-EA.

## 1 INTRODUCTION

Breast Cancer (BC) is one of the most prevalent tumors in women, and according to global cancer statistics, it has an estimated 2.3 million new cases (11.7%) [1]. The incidence of female BC in China has remained high since 2000, accounting for 18% of female BC worldwide [2]. Estrogen Receptor (ER) plays an important role in the progression of BC. There are two subtypes of ER, ERα and ERβ, with differential expression and function in BC. Studies have reported that about 2/3 of ER^+^BCs present high expression levels of ERα^+^ at initial diagnosis [3]. High expression of ERα and activation of signaling pathways are major drivers of ER^+^BC.

ER-independent pathway has been identified as the primary mechanism of BC induction [4]. The PI3K/Akt signaling pathway is critical in the BC cell cycle progression, proliferation, and apoptosis [4, 5]. Extracellular ligands activate PI3K, which recruits two protein kinases, Akt and PDK1, to the plasma membrane. Additionally, the ligands phosphorylate the kinases through their pleckstrin homology interaction domain [6]. Activated Akt phosphorylates target proteins on the cell membrane and detaches from the membrane to phosphorylate other target proteins in the cytoplasm and nucleus, stimulating cell survival, growth, and proliferation [7-9].

The cell cycle is divided into the G0/G1 phase, S phase, and G2/M phase, and cell cycle regulation is the primary mechanism that determines a series of physiological processes, such as cell proliferation and differentiation, as in the G1-S phase by binding to CDK with Cyclin B1 and Cyclin D1 [10]. The expression of cell cycle proteins is also regulated by the upstream PI3K/Akt pathway [11]. Akt can mediate cell survival by directly inhibiting p21 and p27 expressions [12, 13], thus negatively regulating cell cycle progression. Therefore, the ERα pathway and PI3K/Akt pathway mediate the proliferation and apoptosis of BC cells, which are the key regulatory pathways in the development of ER^+^BC, and the comprehensive inhibition of ERα and PI3K/Akt signaling pathways is an important strategy for anti-ER^+^ BC [14].

Clinical studies have reported that Traditional Chinese Medicine (TCM) can effectively prolong the survival rate and improve the quality of survival of breast cancer patients [15-20]. *Amorphophalli rhizoma* (APR) is commonly used in China as a clinically characteristic anticancer TCM that has the potential against breast cancer [21]. Studies have reported that APR could inhibit cell proliferation and metastasis in triple-negative BC [22-24]. The petroleum ether and ethyl acetate extracts of APR acted on triple-negative BC MDA-MB-231 cells, thus causing arrest in the S phase [23]. This drug-containing serum effectively inhibited cell proliferation and suppressed breast cancer metastasis in mice lungs [24]. Another study revealed that APR-extract significantly depressed MDA-MB-231 cell proliferation, migration, and invasion by inhibiting the PI3K/Akt signaling pathway [22]. Different extracts of APR have shown good therapeutic efficacy against BC. However, there has been no systematic investigation into the effect of APR and its various extracts on the estrogen receptor-positive (ER^+^) BC model.

Thus, the aim and objectives of the present study were to investigate the antiproliferative effects of different extracts of APR (petroleum ether, ethyl acetate, n-butanol, and water) on ER+ T47D breast cancer cell lines. Based on the identified effective concentration, we examined the anti-invasive and migratory effects using transwell and cell scratch assays. Finally, we investigated the molecular mechanism of ethyl acetate extract of APR using the ER^+^ BC breast cancer cell line.

## 2 MATERIALS AND METHODS

### 2.1 Chemicals and Reagents

*Amorphophalli rhizoma* (APR) (Lot. 2004192) was purchased from Zhejiang Traditional Chinese Medicine Hospital. Ethanol 95% (Lot. 20190130), petroleum ether (Lot. 20181126), ethyl acetate (Lot. 20190402), and n-butanol (Lot. 20190511) were purchased from Shuanglin Inorganic Chemical Plant, Shanghai Lingfeng Chemical Reagents Co., Ltd. T47D cell line (Lot.CTCC-400-0233) was purchased from Hangzhou Xin ran Biotechnology Co. Ltd. DMEM medium (Lot. 06-1055-57-1ACS) was purchased from Biological Industries, Israel. Cyanostreptavidin solution (100×) (Lot. GNM15140) was purchased from Genom Biomedical Technology Co. Ltd. Fetal Bovine Serum (FBS) (Lot. SA212.02) was purchased from Cellman, Switzerland. PBS (Lot. SH30256.01) was purchased from HyClone Corporation, USA. CCK8 kit (Lot. BS350B) was purchased from Beijing Labgic Technology Co., Ltd. Matrigel Matrix Gel (Lot. 356234) and Transwell (Lot. 3422) were purchased from Corning, USA. Crystalline Violet (Lot. BS941-5g) and 4% paraformaldehyde (Lot. BL539A) were purchased from Beijing Labgic Technology Co., Ltd. Antibodies, including PI3K (Lot.4249T), p-PI3K (Lot.4228T), Akt (Lot. 4691T), p-Akt (Lot. 4060T), Bax (Lot. 5023T), Bcl2 (Lot. 4223T), P21 (Lot. 2972S), ERα (Lot. 8644T), and β-actin (Lot. 4967S) Rabbit mAb were purchased from CST Corporation, USA. CDK4 (Lot. A16813) Rabbit mAb was purchased from Wuhan Ai Bo Taco Biological Technology Co. Ltd.

### 2.2 Preparation of Different APR Extracts

We weighed the dried medicinal slices of APR, crushed them into coarse particles, poured them into a 10 L round-bottomed flask, and soaked them with 95% ethanol. Furthermore, it was placed in an electric heating jacket for heating reflux extraction 3 times, each for 1 hour. Filtration was sucked to collect the filtrate; extract collection was done thrice and concentrated under reduced pressure. It was recycled without alcoholic flavor, and the residue was diluted with distilled water (APR-WAT).

The aqueous suspension was extracted with petroleum ether several times using the systematic solvent method, and the combined extracts were concentrated under reduced pressure. The extract was dried to obtain petroleum ether extract of *Amorphophalli rhizoma* (APR-PEE). The residue after petroleum ether extraction was further extracted with ethyl acetate by systematic solvent method several times, and the collected extract was concentrated under reduced pressure and dried to obtain ethyl acetate extract of *Amorphophalli rhizoma* (APR-EAE). The residue after ethyl acetate extraction was further extracted with n-butanol by systematic solvent method several times, and the obtained extract was concentrated under reduced pressure and dried to obtain n-butanol extract of *Amorphophalli rhizoma* (APR-NBE). APR-PEE, APR-EAE, and APR-NBE were dissolved in DMSO and filtered after full dissolution; a final concentration of 100 mg/mL was prepared and stored in the refrigerator at 4°C for use.

### 2.3 Phytochemical Analysis of APR Extract

#### 2.3.1 Phytochemicals Detection by Ultra High-Performance Liquid Chromatography (UHPLC)

In 100 µL of water extract, 300 µL methanol was added, and vortex mixing was done for 10 min, centrifuged at 13000 rpm for 10 min, and the supernatant was taken for analysis. Then, 0.1 g of ethyl acetate (EA) extract was weighed, 1 mL methanol was added, grinding beads were added for 5 min, mixed through vertexing for 10 min, centrifuged at 13000 rpm for 10 min, and the supernatant was taken for analysis.

### 2.4 Cells and Cell Culture

Human BC T47D (ER^+^) breast cancer cells were obtained from the Shanghai Institute of Cell Biology, Chinese Academy of Sciences (Shanghai, China) and cultured in DMEM supplemented with 10% FBS at 37 ^◦^C in an incubator (Thermo Fisher, MA, USA) containing 5% CO_2_.

### 2.5 Cell Activity Analysis

Cells were inoculated into a 96-well plate. The groups were as follows: blank control group (Veh) (0.1% DMSO concentration), petroleum ether extract of *Amorphophi rhizoma* (APR-PEE) concentration gradient (100, 200, 300, and 400 μg/mL), ethyl acetate extract of *Amorphophi rhizoma* (APR-EAE) concentration gradient (100, 200, 300, and 400 μg/mL), n-butanol extract of *Amorphophi rhizoma* (APR-NBE) concentration gradient (100, 200, 300, and 400 μg/mL), and water extract of *Amorphophalli rhizoma* (APR-WAT). Each group of extraction acted on the cells for 24 h separately. Cell activity analysis was performed using the cell counting kit-8 (CCK-8) method according to established protocols and as already published [22]. In short, 10 μL of CCK8 detection solution was added into each well for 2 h. Each well's optical density (OD) values were read at 450 nm wavelength. The results were calculated according to the following formula:


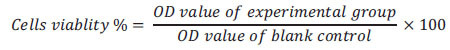


### 2.6 Transwell Experiment

According to our previous study, cell invasion assays were performed using a Transwell chamber with a Matrigel matrix [22]. Briefly, the drugs were grouped as follows: blank cell group, 400 μg/mL APR-PEE, 200 μg/mL APR-EAE, and 400 μg/mL APR-NBE. The lower chamber was added with 600 μL the corresponding medium as the chemotaxis factor so that the liquid surface was submerged in the lower edge of the upper chamber and incubated at 37°C with 5% CO_2_ for 24 h. Then, the upper chamber was washed with PBS, and the Matrigel gel was scraped off with a cotton swab, fixed with 4% paraformaldehyde, stained with 0.5% crystal violet, then left at room temperature for 20 min, and then washed with PBS. The non-migrated cells were wiped clean with a cotton swab. Four 200× field view photographs (200×) of the upper left, upper right, lower left, and lower right were chosen, and the number of their cells was counted to determine the invasive ability of tumor cells.

### 2.7 Wound Healing Assay

The wound-healing assay was performed according to our previous work [22]. Briefly, when the cells were spread all over the well plate and grew to about 90% confluence, the cell supernatant was discarded, the serum-free culture medium was added, and the transverse line perpendicular to the back of the gun tip was used for the cell scratching treatment. The supernatant was discarded, the floating cells were washed with PBS, and the serum-free medium was added. Moreover, before the cells were added, i.e., 0 h, each group was photographed, and the distance between the scratches of each group was recorded, after which the medium containing different concentrations of the drug was added to continue the cultivation. The drugs were grouped as follows: blank cell group, 400 μg/mL APR-PEE, 200 μg/mL APR-EAE extraction site, and 400 μg/mL APR-NBE. After adding the drug and culturing at 37°C, 5% CO_2_ incubator for 24 h and 48 h, respectively, the cells of each group were inverted and observed under the microscope and photographed by AE2000 inverted microscope (200×). The scratch distance was recorded, and the cell migration rate of each group was calculated according to the following formula.


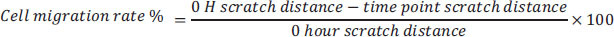


### 2.8 Morphological Observation of Cell Apoptosis

T47D cells with good growth status and in the logarithmic phase of growth were collected and put into cell suspension for cell counting. The cell density was adjusted to 1×10^5^ cells/mL, and the cells were laid on a 96-well plate, where each well was divided into 4 groups, which were further divided into 8 groups. Furthermore, 100 μL of cell suspension was added to each well and put into the incubator for overnight incubation. After the overnight incubation, the cell supernatant was discarded, and the drug was administered in groups: APR-EAE (100, 200, and 400 μg/mL); 0.1% DMSO was also set as a negative control group. The corresponding drug medium was given to 100 μL/well for intervention. After 24 h of action, the morphological changes of the cells were observed and photographed under a high-power microscope. For fluorescence staining, after 24 h of separate interventions, the original medium was aspirated, 1 mL of PBS was added to wash the cells once, and Hoechst 33342 staining solution was added to stain the cells for 10 min, after which the cells were observed under a fluorescence microscope.

### 2.9 Flow Cytometry Detection of Apoptosis Experiment

After 24 h of incubation in the cell culture incubator, the culture medium was discarded, washed with PBS twice, an appropriate amount of 0.25% trypsin was added to digest the cells, and they were incubated at 37 °C. The amount of serum-containing medium was added as trypsin to terminate the digestion, gently blew to let the cells fall off, and then collected the suspended cells directly into 1.5 mL Eppendorf (EP) tubes, with the same operation in each well. Centrifugation was performed for 5 minutes at 1000 rpm/min, and the supernatant in the EP tubes was carefully aspirated to avoid sucking out the cells.

Furthermore, 1×binding buffer was then added to wash once, and the cells were centrifuged at 1000 rpm/min for 5 min; afterward, the supernatant was discarded. A blank tube was set up along with a single staining tube for compensation adjustment. Then, 5 μL of AnnexinV and 5 μL of PI staining solution were added to the sample tubes, respectively. They were kept away from light for 15 min at room temperature before performing flow cytometry. For flow cytometry analysis, FITC and PE channels were used for the detection under 488 nm and 561 nm lasers, respectively.

### 2.10 Flow Cytometry for Cell Cycle Detection Experiment

After 24 h of culture in the cell incubator, pre-cooled 70% ethanol was added to the cells, and they were resuspended sufficiently and fixed in the refrigerator at 4°C overnight. The ethanol was discarded after centrifugation, the cells were washed twice with PBS, the PI/RNase cell cycle staining solution was added, and the cells were stained for 30 min away from light to detect the cell cycle distribution by flow cytometry. The results were analyzed using the cell cycle fitting software Modfit ^TM^.

### 2.11 Western Blotting

The western blotting procedure was performed similarly to the one described previously, with minor changes [22]. The quantity of total loading protein was 1µg. Protein contents were estimated by using BCA protein. A sodium dodecyl sulfate-polyacrylamide gel electrophoresis (SDS-PAGE) was prepared to load and separate the same amount of protein and transfer it to the PVDF membranes. After blocking in 5% skimmed milk at room temperature for 2 h, the culture membrane was incubated with primary antibodies (Bax, Bcl-2, p21, CDK4, ERα, p-PI3K, PI3K, p-Akt, Akt, and β-actin) at 4 °C overnight. Furthermore, enzyme-labeled secondary antibody washing was done at room temperature for 2 h. The protein bands were visualized through ultra-sensitive ECL and quantified using ImageJ software.

### 2.12 Statistical Analysis

All data are presented as mean ± SD. Statistical analysis was carried out using SPSS 17 software in this study. Statistical differences among the groups were assessed using the Student’s T-Test and a one-way analysis of variance (ANOVA). The difference was considered significant if *P* < 0.05. The graphs were designed by using GraphPad Prism 8.0.2 (supplementary material).

## 3 RESULTS

### 3.1 Phytochemicals Detected in *Amorphophalli rhizoma* Ethyl Acetate (APR-EAE) Extract

**Fig. (1):**
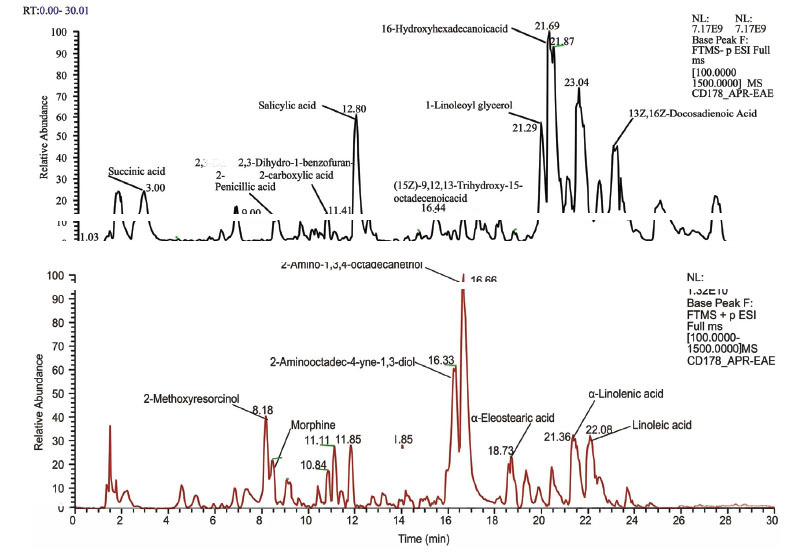
UHPLC-MS Chromatograms of *Amorphophalli rhizoma* (APR-EAE) in (**A**) Positive-Ion Mode and (**B**) Negative-Ion Mode.

Ultra-High-Performance Liquid Chromatography (UHPLC) of the APR-EAE extract revealed the presence of various phytochemicals, such as succinic acid, 2-methoxy resorcinol, penicillic acid, morphine, salicylic acid, α-linolenic acid, and linolenic acid. The UHPLC chromatogram is shown in Figs. **1A** and **B**. The retention time of phytochemicals is given in Table **1**. Phytochemical chemical structures are depicted in Fig. (**2**).

## 4 EFFECTS OF DIFFERENT APR EXTRACTS ON CELL VIABILITY

After 24 hours of treatment administration, the effects of all the extracts (APR-PEE, APR-EAE, APR-NBE, and APR-WAT) on cell viability were compared at various concentrations (100, 200, 300, and 400 µg/mL). The lowest concentration, 100 µg/mL, significantly inhibited cell proliferation in a dose-dependent manner (*p* < 0.001, 0.01) compared to the vehicle group. The most significant inhibitory effects were observed at the concentration of 400 µg/mL for all the extracts, resulting in inhibitions of 55.2%, 57.98%, 34.23%, and 11.2% cell viability, respectively. It is suggested that APR-EAE had the strongest anti-proliferative effect on the breast cancer cell line (T47D), followed by APR-PEE extract. Results are depicted in Table **2**. IC_50_ values were calculated for all the extracts and are presented in Table **3**.

### 4.1 Effect of Different APR Extracts on the Cells Invasion

**Fig. (3):**
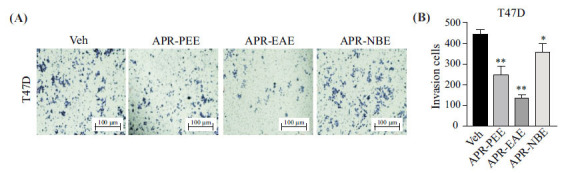
Effects of APR Extracts on Cell Invasion. **(A)** Effects of APR various extracts (APR-PEE, APR-EAE, and APR NBE) on cell invasion assessed through Tanswell assay. **(B)** Quantification of cell invasion with the help of graphical representation. Groups were statistically analyzed by One-way Analysis of Variance (ANOVA). The difference is considered significant if *P* < 0.05. *P < 0.05, **P < 0.01 vs. Veh. **Veh or vehicle**: Blank control group; **APR-PEE**: Petroleum ether extract of *Amorphophalli rhizoma* group (400 μg/mL); **APR-EAE**: Ethyl acetate extract of *Amorphophalli rhizoma* group (200 μg/mL); **APR-NBE:** N-butanol extract of *Amorphophalli rhizoma* group (400 μg/mL). The scale bar was 100 µm.

APR-PEE, APR-EAE, and APR-NBE extracts were further evaluated through transwell assays for their ability to inhibit cell migration and invasion, which is one of the characteristics of breast cancer. Cell invasion was examined after 24 hours of administering the vehicle, and all these extracts were used at various concentrations: APR-EAE 200 μg/mL, APR-EAE 200 μg/mL, and APR-NBE 400 μg/mL. It was found that they significantly (*p* < 0.05, 0.01) inhibited the invasion of BC cell line T47D. The percentage of inhibition was 43.5%, 68.9%, and 18.6%, respectively. The APR-EAE produced the most significant effects on cell invasion, as shown in Figs. **3A** and **B**.

### 4.2 Effect of Different APR Extracts on Cell Migration

**Fig. (4):**
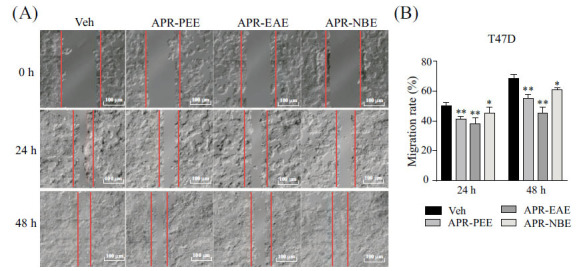
Effects of APR Extracts on Cell Migration. (**A**) The effects of various APR extracts (APR-PEE, APR-EAE, and APR NBE) on cell migration; (**B**) The quantification of cell migration distance with the help of graphical representation. Groups were statistically analyzed using a One-way Analysis of Variance (ANOVA). The difference is considered significant if *P* < 0.05. *P < 0.05, **P<0.01 *vs*. Veh. **Veh or vehicle**: Blank control group; **APR-PEE**: Petroleum ether extract of *Amorphophalli rhizoma* group (400 μg/mL); **APR-EAE**: Ethyl acetate extract of *Amorphophalli rhizoma* group (200 μg/mL); **APR-NBE:** N-butanol extract of *Amorphophalli rhizoma* group (400 μg/mL). The scale bar was 100 µm.

Cell scratch experiments are usually conducted to investigate the migratory ability of the cancer cells. APR-PEE 400 μg/mL, APR-EAE 200 μg/mL, and APR-NBE 400 μg/mL were administered, and the scratch distance of each group was calculated at 0, 24, and 48 hours. After 24 and 48 h of administration, all the extracts mentioned above significantly (*p* < 0.05, 0.01) inhibited cell migration, and the migration rate was 45.2%, 55.5%, and 38.7%, respectively, after 48 hours. APR-EAE had the most potent anti-migration effect in T47D cancer cells, as depicted by cell scratch distance in Fig. **4A**. Graphical quantification is shown in Fig. **4B**.

### 4.3 Effects of APR-EAE on Cell Morphology

**Fig. (5):**
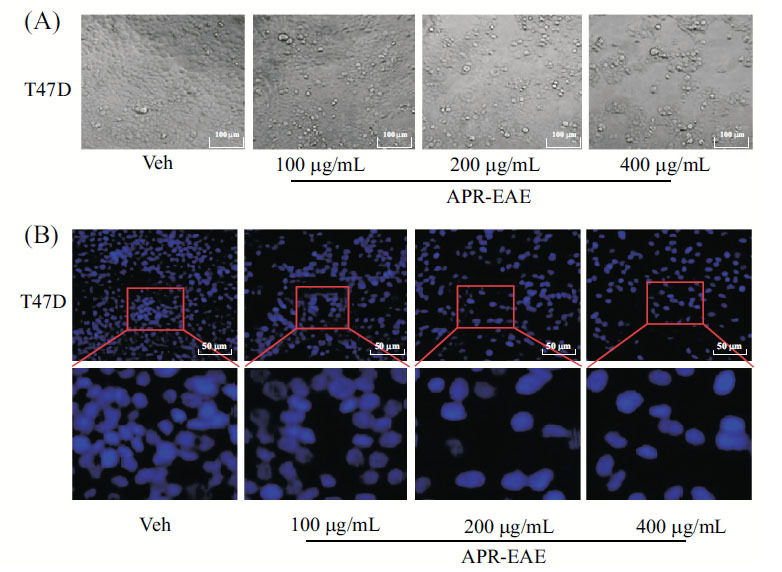
Effects of APR-EAE on Cell Morphological Changes. (**A**) Cell shape under the normal microscope (200X); (**B**) Shape of cell nucleus under the fluorescent microscope (400×). **Veh:** Blank control group; **APR-EAE:** Ethyl acetate extract of *Amorphophalli rhizoma* group.

APR-EAE concentrations of 100, 200, and 400 μg/mL were applied to the breast cancer cell line (T47D) for 24 h. Compared to the vehicle control group, the changes in cell morphology showed a pathological nature. The connection between the cells gradually disappeared, and the cell volume began to shrink. With the increasing concentrations of APR-EAE, the cell morphological changes became more apparent at the highest concentration, showing a typical pattern of dose dependence (Fig. **5A**).

Furthermore, Hoechst 33342 staining was applied, and microscopic pictures revealed that APR-EAE induced apoptotic morphological changes in breast cancer cells, as shown in Fig. **5B**. Florescent microscopy showed the nuclei of cells in the administered group bright blue, and some nuclei were lobulated or fragmented. At the same time, this phenomenon was not observed in the control group. As the APR-EAE concentration increased, the cell volume gradually reduced. The cytoplasmic density increased, the nucleus and nucleoplasm appeared condensed, the nuclear membrane and nucleolus were gradually fragmented, and apoptotic vesicles eventually formed. It was suggested that APR-EAE could promote apoptosis in these cells in a dose-dependent manner.

### 4.4 Effects of APR-EAE on Cell Apoptosis

**Fig. (6):**
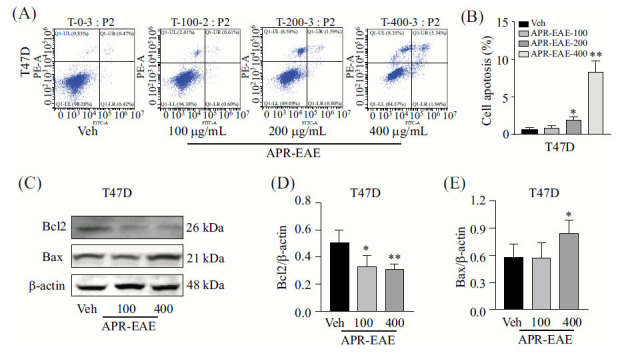
Effects of APR-EAE on Apoptosis. (**A**) Flow cytometry chart of apoptosis in breast cancer cell line T47D. (**B**) Apoptotic cell quantification with the help of a graph. (**C**) Western blot images of the expression pattern of Bcl2 and Bax proteins along with β-actin. (**D**) Brandwidth quantification of Bcl2 (**D**) Bax protein. Groups were statistically analyzed by one-way analysis of variance (ANOVA). The difference is considered significant if *P* < 0.05. *P < 0.05, **P<0.01 vs veh. Veh: Blank control group; APR-EAE: Ethyl acetate extract of *Amorphophalli rhizoma* group.

To further observe the pro-apoptotic effect of APR-EAE on the BC cell line (T47D), the apoptotic rate was determined by flow cytometry using Annexin VFITC/PI double staining. The proportion of apoptotic cells gradually increased with the increase in APR-EAE concentration. The apoptosis rate of the T47D cell line was 0.86% at a concentration of 100 μg/mL, while it reached 8.32% after 400 μg/mL treatment. It is evident that the rate of apoptosis was concentration-dependent, as shown in Figs. (**6A** and **B**). The breast cancer cell line (T47D) was treated with APR-EAE concentrations of 100 and 400 μg/mL for 24 h, and it was found that apoptosis check pint protein Bcl2 was significantly down-regulated and apoptosis initiating protein Bax was significantly up-regulated. It was suggested that APR-EAE induced apoptosis by regulating Bax/Bcl2 (Figs. **6C**-**D**).

### 4.5 Effects of APR-EAE on the Cell Cycle

We extended our work to determine the effect of APR-EAE on the cell cycle stages, and flow cytometry was used to determine the cell cycle stages. Compared to the vehicle group, APR-EAE 100, 200, and 400 μg/mL significantly (*p* < 0.01) increased the percentage of the G0/G stage. Similarly, 200 and 400 μg/mL increased the percentage of the G2/M phase and reduced the cell cycle's S-phase ratio. After treatment with APR-EAE 100 μg/mL, the percentage of total cells in the G0/G1, G2/M, and S phases was 59.94%, 10.14%, and 29.92%, respectively, compared to the vehicle group. After treatment with 200 µg/mL, this percentage reached 59.46%, 12.65%, and 27.89, and after treatment of 400 µg/mL, this percentage was 60.81%, 16.99%, and 22.20%. Thus, these findings indicated that the percentage of cells increased significantly (*p* < 0.01) in the G0/G1 phase and G2/M and decreased in the S phase.

The percentage of total T47D cells in G0/G1, G2/M, and S phases after 200 μg/mL treatment was 59.46%, 12.65%, and 27.89%; the percentage of total T47D cells in G0/G1, G2/M, and S phase after 400 μg/mL treatment was 60.81%, 16.99%, and 22.20% respectively. This indicated that APR-EAE significantly increased the number of T47D cells in the G0/G1 phase and G2/M phase and decreased the percentage of cells in the S phase. Results are shown in Figs. (**7A**-**B**).

**Fig. (7):**
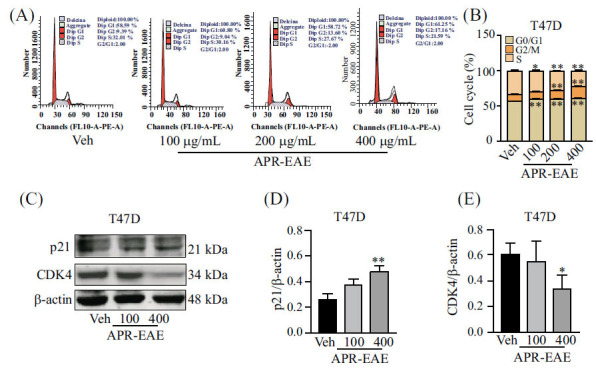
Effects of APR-EAE on the Cell Cycle. (**A**) The effects of APR EAE on cell cycle stages are shown in the flow cytometry chart. (**B**) Quantification of the cell number in various stages of the cell cycle. (**C**) Protein expression detected by western blot. (**D**) P21 protein expression band quantification was relative to housekeeping protein β-actin. (**E**) CDK4 protein expression band quantification relative to housekeeping protein β-actin. Groups were statistically analyzed using a one-way analysis of variance (ANOVA). The difference is considered significant if *P* < 0.05. *P < 0.05, **P < 0.01 *vs.* veh. Veh: Blank control group; APR-EAE: Ethyl acetate extract of *Amorphophalli rhizoma* group.

Additionally, we determined the level of cell cycle regulatory proteins, such as P21 and CDK10, with the help of Western blot. P21 is a negative regular of the cell cycle, while CDK10 is a positive regulator. Our study findings revealed that APR-EAE 400 μg/mL significantly (*p* < 0.05, 0.01) increased the level of p21 protein and significantly decreased the level of CDK4 protein in the T47D breast cancer cell line compared to the vehicle control group. Thus, it is apparent that APR-EAE could inhibit the downstream expression of cyclic CDK4 protein and increase the expression of cyclic P21 protein (Figs. **7C**, **D**, and **E**).

### 4.6 Effects of APR-EAE on Estrogen Receptor α (ERα) and Phosphatidylinositol 3-Kinase/Protein Kinase B (PI3K/Akt) Signaling Pathway in Breast Cancer

**Fig. (8):**
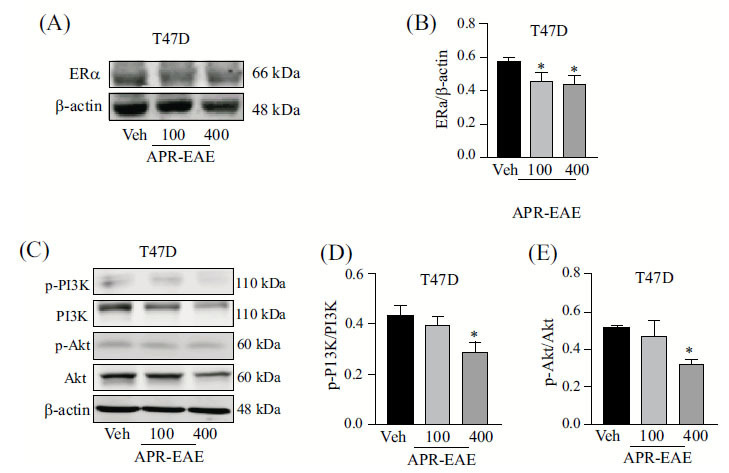
Effects of APR-EAE on ERα and PI3K/Akt Signaling Pathway in Breast Cancer. (**A**) Western blot images of protein expression Erα in breast cancer cell line T47D. (**B**) Quantification of band density of Erα/β-actin. (**C**) Western blot images of protein expression of p-PI3K, PI3K p-Akt, and Akt in breast cancer cell line T47D. (**D**) Quantification of band density of p-PI3K/PI3K. (**E**) p-Akt/Akt protein relative to β-actin. Groups were statistically analyzed by one-way analysis of variance (ANOVA). The difference is considered significant if *P* < 0.05. *P < 0.05, **P < 0.01 *vs.* veh. Veh: Blank control group; APR-EAE: Ethyl acetate extract of *Amorphophalli rhizoma* group.

We determined the effect of APR-EAE 9100 and 400 μg/mL on the ERα and found that it significantly (*P* < 0.05, 0.01) downregulated the expression of ERα protein expression in a dose-dependent manner. The results are shown in Figs. **8A**-**B**. We were interested in determining the signaling pathway involvement behind the effects of APR-EAE. Our study findings revealed that APR-EAE significantly (*P* < 0.05, 0.01) down-regulated p-PI3K/PI3K and p-Akt/Akt protein expression in breast cancer cell line (T47D). Therefore, these findings indicated that APR-EAE could inhibit the PI3K/Akt signaling pathway and ERα high expression in ER^+^ breast cancer cell line T47D (Figs. **8C**, **D**, and **E**).

## 5 DISCUSSION

*Amorphophalli rhizoma* (APR) is a Traditional Chinese Medicine (TCM). It has been used traditionally for the treatment of cancer for a long time. Traditional Chinese Medicines (TCM) have been found effective in halting tumor reoccurrence and metastasis. APR-containing preparations were found effective in improving clinical symptoms and could prolong the survival time of cancer patients [22, 23]. Recent research has shown that ethyl acetate extract of APR has better outcomes in *in vitro* analysis [25]. Another study reported that petroleum ether extract and ethyl acetate extract of APR could block gastric cancer cells in the G0/G1 phase and induce apoptosis with a specific dose-dependent effect [26]. They also significantly inhibited the proliferation of MDA-MB-231 cells in triple-negative BC *in vitro* [23], and it was found that the petroleum ether extract of APR could inhibit the proliferation, migration, invasion, and metastasis of MDA-MB-231 cells [22]. APR might exert anti-tumor effects on BC and other tumors through multi-components, in which ethyl acetate and petroleum ether extract may be essential sites to play a role.

Breast cancer cells have a rapid progression [27]. Therefore, it is very important to determine the effects of treatment on cell proliferation with the help of a valid method. Thus, such assays are very important in cancer research, as they can determine cell proliferation and the effects of treatment on cell progression and proliferation [28, 29]. We used epithelial cell-based ER^+^ breast cancer (BC) cell lines T47D, which have a broader utility in the study of estrogen receptor-positive breast cancer. The effect of APR on breast cancer cell proliferation was assessed by the cell counting kit (CCK-8) method. Various extracts of APR (APR-PEE, APR-EAE, APR-NBE, and APR-WAT) were used to treat ER^+^ breast cancer cells (T47D) for 24 hours. The study findings revealed that APR-EAE was the most effective in inhibiting cell proliferation, up to 57.98% in ER^+^ BC cells (Fig. **3**).

After proliferation, the 2^nd^ most important characteristic of cancer cells is metastasis, which is one of the major causes of death from malignant tumors [30, 31]. It was found that 66.7% of deaths in patients with solid tumors were caused by recurrent metastasis [32]. Generally, BC is often diagnosed after metastasis and has a poor prognosis because it starts with a localized disease and can spread to lymph nodes or distant organs, thus severely hampering the treatment of BC [33].

In the current study, we used cell scratch and Transwell assays to evaluate the anti-migratory and anti-invasion ability of different APR extracts on ER^+^ BC cell lines (T47D). The study results showed that APR-EAE (200 µg/mL) was the most effective among all the other extracts, and it inhibited cell migration by up to 55.55% during cell scratch experiments. Cell scratch experiment results were visualized after 24 and 48 hours (Fig. **4**). In this project, we selected three different extracts of APR: APR-PEE, APR-EAE, APR-NBE, and APR-WAT. The effects of all these extracts were observed on cell proliferation, migration, and invasion, and we found that APR-EAE was the most effective. Our findings are similar to another study, which used APR-EAE on liver cancer cells and found it to be the most effective [34, 35]. Another study also found its effectiveness against the human gastric cancer cell line (SGC-790) [35].

Apoptosis is programmed cell death, and it is a valuable tool to check on cells during the cell cycle or after that to induce programmed death in the cells that undergo pathological abnormalities or some severe damage occurs in their DNA [36-38, 39]. Morphologically, apoptotic cellular changes include cellular sequestration (nuclear condensation and shrinkage), chromatin condensation, sequestration, and the formation of apoptotic vesicles containing cytoplasm, organelles, and nuclear debris [40]. APR-EAE treatment in ER^+^ BC cell line (T47D) for 24 h showed few morphological changes, such as the cellular connections gradually disappearing, the cell volume solidifying, and the cellular morphology changing in a dose-dependent manner, with higher concentrations causing more morphological abnormalities. Similarly, some nuclei, the nuclear membrane, and the nucleolus were gradually fragmented (Fig. **5**). Flow cytometry revealed that APR-EAE significantly (*p* < 0.05) increased the percentage of cells in the G0/G1 phase. Thus, it was evident that it induced apoptosis in the BC ER^+^ cell line (Figs. **6A**-**B**).

During the cell cycle, the Bax/Bcl-2 family proteins regulate apoptosis by controlling pro- and anti-apoptotic intracellular signals. Pro-apoptotic factors, such as Bax, can induce apoptosis by producing mitochondrial pores responsible for MOMP [41]. In contrast, anti-apoptotic factors, such as Bcl-2, can inhibit apoptosis by blocking pro-apoptotic family members [42]. Our study findings showed that APR-EAE treatment (24 hours) down-regulated Bcl-2 and up-regulated Bax protein expression. Thus, APR-EAE induced apoptosis by regulating the Bax/Bcl-2 protein expression (Figs. **6C**, **D**, and **E**. Our study findings were consistent with other research published on human gastric ulcers [35].

Cyclin-Dependent Kinases (CDKs) belong to the serine/threonine kinase family and play a key role in various signaling pathways that regulate transcription and cell cycle progression through their involvement in G1, S, G2, and M phase cellular activities [43]. CDK4/6 inhibitors block suspected tumor cells in the G1 phase of the cell cycle. P21 is a tumor suppressor gene and an important cell cycle inhibitor with kinase activity, which exerts tumor growth inhibitory effects, mainly negatively regulating the cell cycle, and can lead to apoptosis that inhibits tumorigenesis and cancer development [44]. In our study, flow cytometric analysis revealed that APR-EAE treatment significantly increased the proportion of cells in the G0/G1 phases of the cell cycle (Figs. **7A**-**B**). Consistently, Western Blot (WB) analysis showed increased cellular p21 while decreased CDK4 protein expression (Fig. **7C**, **D** and **E**). These changes indicated cell division stoppage, quiescence, and apoptosis initiation in the cells carrying any drastic morphological changes.

The literature review emphasized that ˃ 65% of ER^+^ cells had higher expression of Erα at the time of diagnosis [45]. ERβ might have a lower affinity with E2 receptors compared to ERα, and studies on ER (ERα and ERβ) knockout mice have already reported that ERα plays an essential role in estrogen-mediated metabolic regulation. In contrast, ERβ mice exhibited only mild metabolic regulation phenotyping [46]. Tamoxifen is used to prevent and control the development of BC by inhibiting the estrogen signaling pathway, thus inhibiting the activation of estrogen signaling into the nucleus and regulating the transcription of downstream target genes, which control the proliferation, migration, and apoptosis of breast cancer cells [4]. Our study findings indicated that 24-hour treatment of APR-EAE significantly reduced the expression of Erα in ER^+^ breast cancer cells, highlighting the role of APR-EAE regulatory effects on ERα and its mediated pathways.

The PI3K/Akt signaling pathway is one of the key intracellular signaling pathways and is closely related to various cellular processes, including proliferation, metabolism, and angiogenesis [47]. Two pathways have the most important role in the mechanism of induction of breast cancer: the ER-dependent pathway and the ER-independent pathway [4]. The ER-dependent pathway mainly involves estrogen binding to ERα, which mediates the transcription of target genes downstream of the estrogen signaling pathway in the cell's nucleus to promote BC progression. The ER non-dependent pathway mainly involves the interaction between estrogen signaling pathway and Epidermal Growth Factor Receptor (EGFR)/Human Epidermal Growth Factor Receptor 2 (HER2) signaling pathway, which mainly manifests itself in the activation of various intracellular signaling pathways, such as PI3K/Akt, etc. Moreover, the PI3K/Akt signaling pathway is the most critical pathway, which is involved in BC cell cycle progression, cell proliferation, etc. [4]. It is also the most important cause of poor treatment prognosis in ER^+^ BC.

It was found that PI3K activation was detectable in most BC patients, and PI3K/Akt signaling activation was found to be positively associated with poor prognosis and survival rate [48]. Furthermore, the inhibition of the PI3K/Akt signaling pathway significantly induced apoptosis in BC cells, as demonstrated by *in vitro* experiments [49]. Additionally, the inhibition of the PI3K/Akt signaling pathway can affect the cancer cell cycle and exert anti-BC effects [50]. Thus, the inhibition of the PI3K/Akt signaling pathway is an important strategy for preventing and treating breast cancer. Our study findings also showed that APR-EAE significantly down-regulated P-PI3K/PI3K and P-Akt/Akt protein levels in the ER+ breast cancer (T47D) cell line (Fig. **8**).

## CONCLUSION

In conclusion, the ethyl acetate extract of *Amorphophallus rhizoma* (APR-EAE) effectively inhibited the proliferation, migration, and invasiveness of ER^+^ breast cancer cells. This action might be mediated by the suppression of the PI3K/Akt signaling pathway and the downregulation of ERα expression. These changes likely lead to a decrease in the expression of CDK4 and Bcl-2 proteins while increasing the expression of p21 and Bax proteins. The phytochemicals present in APR-EAE may play a key role in its anticancer effects.

Although APR-EAE showed promise as a complementary treatment for ER^+^ breast cancer, more research is needed to fully understand its therapeutic potential, side effects, and interactions with existing therapies. Challenges in extraction, purification, and consistent dosing hinder its widespread clinical use. Rigorous clinical trials are essential to confirm its safety, efficacy, and role in combination with current treatments for ER^+^ BC.

## Figures and Tables

**Fig. (2):**
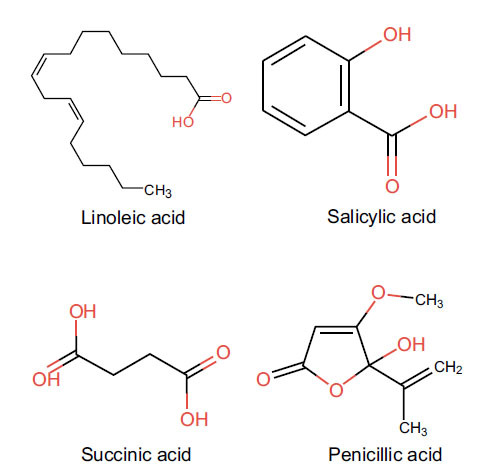
Phytochemical Detected through UHPLC in *Amorphophalli rhizoma* (APR-EAE).

**Table 1:** Phytochemicals Detected in *Amorphophalli rhizoma* (APR-EAE).

**Name**	**Formula**	**m/z**	**RT [min]**	**Relative content (%)**
Succinic acid	C_4_H_6_O_4_	117.01794	3.00	2.06
2-Methoxyresorcinol	C_7_H_8_O_3_	141.05455	8.181	2.26
Morphine	C_17_H_19_NO_3_	286.14359	8.486	1.02
Penicillic acid	C_8_H_10_O_4_	171.06522	9.097	0.65
2,3-Dihydro-1-benzofuran-2-carboxylic acid	C_9_H_8_O_3_	163.039	11.412	0.38
Salicylic acid	C_7_H_6_O_3_	137.02309	12.804	2.49
2-Aminooctadec-4-yne-1,3-diol	C_18_H_35_NO_2_	298.27344	16.305	4.46
(15Z)-9,12,13-Trihydroxy-15-octadecenoic acid	C_18_H_34_O_5_	329.23318	16.447	0.74
2-Amino-1,3,4-octadecanetriol	C_18_H_39_NO_3_	318.29959	16.669	8.80
α-Eleostearic acid	C_18_H_30_O_2_	279.23157	18.748	0.51
1-Linoleoylglycerol	C_21_H_38_O_4_	355.28348	21.291	0.45
α-Linolenic acid	C_18_H_30_O_2_	279.23141	21.361	0.73
16-Hydroxyhexadecanoic acid	C_16_H_32_O_3_	271.22742	21.696	6.12
Linoleic acid	C_18_H_32_O_2_	279.23273	22.086	0.66

**Table 2:** Effects of various extracts of APR on T47D cell viability.

**Concentration (μg/mL)**	**Cell Viability(%)**			
	**APR-PEE**	**APR-EAE**	**APR-NBE**	**APR-WAT**
Veh	100±5.48	100±0.43	100±1.75	100±2.39
100	86.10±1.36**	73.01±3.25***	89.37±2.71***	93.41±0.50**
200	70.7±1.79***	51.90±1.41***	75.75±2.30***	92.07±1.11**
300	57.84±1.30***	49.21±3.35***	71.37±0.92***	90.75±0.54**
400	44.78±1.54***	42.01±1.52***	65.77±1.80***	88.83±1.14***

Note:*P < 0.05, **P < 0.01, ***P < 0.001 vs. Vehicle group. n=6.

**Table 3:** IC_50_ Value of various APR extract of T47D cell viability.

**Groups**	**IC_50_ µg/mL**
APR-PEE	˃ 400
APR-EAE	110.5 ± 12
APR-NBE	220.1 ± 164.2
APR-WAT	˃ 400

## Data Availability

The data used to support the findings of this study are available from the corresponding author upon reasonable request.

## LIST OF ABBREVIATIONS

AKT: Serine/threonine Kinase
APR: *Amorphophalli rhizoma*
APR-EAE: *Amorphophalli Rhizoma* Ethyl Acetate Extract
APR-NBE: *Amorphophalli Rhizoma* n-Butanol Extract
APR-PEE: *Amorphophalli Rhizoma* Petroleum Ether Extract
APR-WAT: *Amorphophalli Rhizoma* Water Extract
Bax: *BCL2-associated X protein*
BC: Breast Cancer
Bcl2: B-cell lymphoma-2
CDK4: Cyclin-Dependent Kinases 4
ER^+^: Estrogen Receptor-Positive
P21: Cyclin-dependent kinase inhibitor 1A
PI3K: Phosphatidylinositol 3 kinase
TCM: Traditional Chinese Medicine

## SUPPLEMENTARY MATERIAL

Data related to the UHPLC materials and methodology are included in supporting information.
