# Supplementary material for: Pharmacological Evaluation of Amorphophalli rhizoma to Inhibit the Progression of Estrogen Receptor+ (ER+) Breast Cancer by Modulating the PI3K/AKT Cell Signaling Pathway
Source: Curr Cancer Drug Targets. 2025 Mar 5;26(2):162–75. doi: 10.2174/0115680096360994250106082347 (PMC13284660; doi:10.2174/0115680096360994250106082347)
Supplement: Supplementary file 1 — Data related to the UHPLC materials and methodology are included in supporting information. [file CCDT-26-2-162_SD1.pdf]

Supplementary Material

Pharmacological Evaluation of *Amorphophalli rhizoma* to Inhibit the Progression of Estrogen Receptor$^+$ (ER$^+$) Breast Cancer by Modulating the PI3K/AKT Cell Signaling Pathway

Hailong Li[1], Qinghong Yu[2], Jiaqing Song[2], Haining Ding[2], Yian Chen[2], Ying Jin[2], Hongting Wu[2], Liaqat Hussain[3,*] and Xiufei Gao[4,*]

[1]School of Green Intelligent Pharmaceutical, Zhejiang Guangsha Vocational and Technical University of Construction, Dongyang, Zhejiang, China; [2]First Clinical Medical College, Zhejiang Chinese Medical University, Hangzhou, Zhejiang, China; [3]Department of Pharmacology, Faculty of Pharmaceutical Sciences, Government College University, Faisalabad, Pakistan; [4]Department of Breast Surgery, The First Affiliated Hospital of Zhejiang Chinese Medical University, Zhejiang Provincial Hospital of Chinese Medicine, Hangzhou, Zhejiang, China

1. PHYTOCHEMICALS DETECTION BY ULTRA HIGH-PERFORMANCE LIQUID CHROMATOGRAPHY (UHPLC)

1.1. Materials, Equipment, and Methods

Methanol: chromatographic grade, Thermo Fisher Scientific (China) Co., Ltd. Formic acid: chromatographic grade, Shanghai Aladdin Biochemical Technology Co., Ltd.

Polypropylene centrifuge tube: 1.5mL, 2.0mL, Wuhan Sevier Biotechnology Co., Ltd. Pure water/ultrapure water integrated system: Direct-Q®5, Merck Millipore, Germany

High-speed micro-refrigerated centrifuge: D3024R, Beijing Dalong Xingchuang Experimental Instrument Co., Ltd. Vortex oscillator: MX-F, Wuhan Sevier Biotechnology Co., Ltd.

Ultrasonic cleaner: JP-040S, Shenzhen Jiemeng Cleaning Equipment Co., Ltd.

Pipette: 2.0~20.0 µL, 20.0~200 µL, 200~1000 µL, Eppendorf

Chromatograph: UltiMate 3000 RS, Thermo Fisher Scientific (China) Co., Ltd.

Mass spectrometer: Q Exactive high-resolution mass spectrometer, Thermo Fisher Scientific (China) Co., Ltd.

1.2. Method or Procedure

In water extract 100 µL, added 300 µL methanol, vortex mixing was done for 10 min, centrifuged at 13000 rpm for 10 min, and the supernatant was taken for analysis. 0.1 g of ethyl acetate (EA) extract was weighed, 1 mL methanol, was added grinding beads for 5 min, mixed on vortex for 10 min, centrifuged at 13000 rpm for 10 min, and supernatant was taken for analysis. The detection was done with mass spectrometric conditions; ion source was an electrospray ionization source (ESI). The chromatographic gradient is shown in Table S1, and matched compounds are shown in Table S2.

The following detection and spectrophotometric conditions were maintained as:

Ion source: electrospray ionization source (ESI) Scan mode: positive and negative ion switching Scan detection mode: Full mass/dd-MS2

Resolution: 70000 (full mass); 17500 (dd-MS2) Scan range: 100.0～1500.0 m/z

Electrospray voltage: 3.2 kV (Positive, Negative) Capillary temperature: 300 °C

Collision gas: high purity argon (purity ≥ 99.999%) Collision energy (N) CE: 30, 40, 60, Sheath gas: nitrogen (purity ≥ 99.999%), 40 Arb, Auxiliary gas: nitrogen (purity ≥ 99.999%), 15 Arb, 350 °C, Data acquisition time: 30.0 min

Chromatographic conditions: Chromatographic column: AQ-C18, 150×2.1mm, 1.8 µm, Welch

Flow rate: 0.30 mL/min

Aqueous phase: 0.1% formic acid/water solution Organic phase: methanol

Column oven temperature: 35°C

Autosampler temperature : 10.0°C

Autosampler injection volume : 5.00 µL

Table S1: Chromatographic Gradient

Time (min.)	Aqueous phase ratio (%)	Organic phase ratio (%)
1	98	2
5	80	20
10	50	50
15	20	80
20	5	95
27	5	95
28	98	2
30	98	2

The data collected by high-resolution LC-MS was sorted by CD 3.3 (Compound Discoverer 3.3) (Thermo Fisher) and then searched and compared with the database (mz Cloud).

Table S2: Compounds Matched

Sample	Compounds matched in mz Cloud	Comprehensive score greater than 60 points in mz Cloud best match
ARW	689	326
AWE	1155	583

2. ORIGINAL WESTERN BLOT IMAGES/ RAW DATA

Figures S1, S2, and S3 showed the original western blot image data used in Figures 6, 7, and 8, respectively.

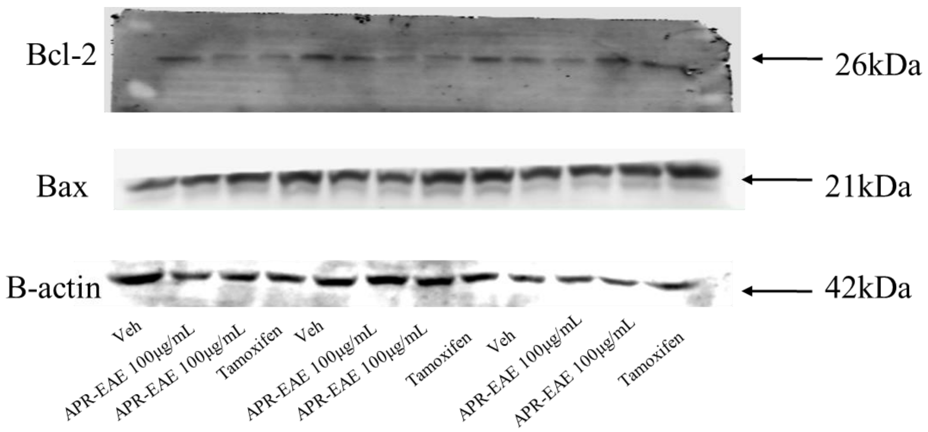

Figure S1: The original western blot data used in Figure 6.

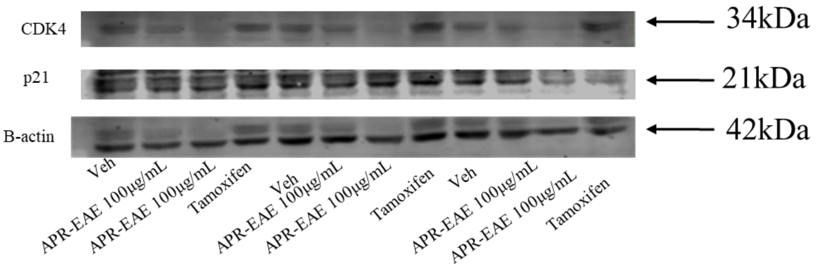

Figure S2: The original western blot data used in Figure 7.

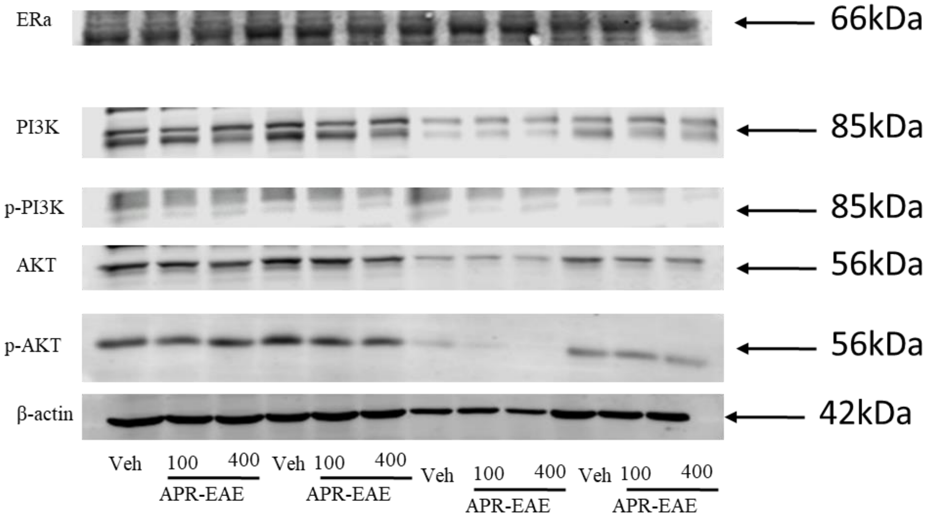

Figure S3: The original western blot data used in Figure 8.
